# Reflection on modern methods: shared-parameter models for longitudinal studies with missing data

**DOI:** 10.1093/ije/dyab086

**Published:** 2021-06-11

**Authors:** Michael E Griswold, Rajesh Talluri, Xiaoqian Zhu, Dan Su, Jonathan Tingle, Rebecca F Gottesman, Jennifer Deal, Andreea M Rawlings, Thomas H Mosley, B Gwen Windham, Karen Bandeen-Roche

**Affiliations:** 1 The MIND Center, University of Mississippi Medical Center, 2500 N State Street, Jackson, MS, 39216, USA; 2 Department of Data Science, University of Mississippi Medical Center, Jackson, MS, USA; 3 Department of Neurology, School of Medicine, Johns Hopkins University, Baltimore, MD, USA; 4 Department of Epidemiology, Johns Hopkins University, Baltimore, MD, USA; 5 Department of Biostatistics, Johns Hopkins Bloomberg School of Public Health, Johns Hopkins University, Baltimore, MD, USA

**Keywords:** Missing data, joint models, shared-parameter models, sensitivity analyses, informative missingness, missing not at random, censoring, dropout, longitudinal data, reproducible research

## Abstract

A primary goal of longitudinal studies is to examine trends over time. Reported results from these studies often depend on strong, unverifiable assumptions about the missing data. Whereas the risk of substantial bias from missing data is widely known, analyses exploring missing-data influences are commonly done either *ad hoc* or not at all. This article outlines one of the three primary recognized approaches for examining missing-data effects that could be more widely used, i.e. the shared-parameter model (SPM), and explains its purpose, use, limitations and extensions. We additionally provide synthetic data and reproducible research code for running SPMs in SAS, Stata and R programming languages to facilitate their use in practice and for teaching purposes in epidemiology, biostatistics, data science and related fields. Our goals are to increase understanding and use of these methods by providing introductions to the concepts and access to helpful tools.

**Key Messages**

- Results from standard longitudinal modelling approaches such as generalized estimating equations (GEEs) and mixed models can be biased when outcome data are informatively missing.
- Shared-parameter models (SPMs) provide a flexible framework for exploring potential missing-data effects and can be particularly helpful when there are clear connections between longitudinal outcome measurements and related censoring events.
- SPMs can be generalized into larger sensitivity-analysis frameworks for examining untestable assumptions of informatively missing data. SPMs can be estimated using existing software (reproducible Stata, SAS & R code provided).

## Introduction

Missing outcome data occur frequently in longitudinal studies^1^ and even moderate amounts can strongly bias study results.^2^ Whereas significant progress has been made in approaches for handling missing data,^3–5^ regular implementation has been slow to follow^6^ and improper handling of missing data remains one of the biggest concerns in peer-reviewed literature.^7^

Missing and ‘coarsened’ outcome data may arise from a variety of mechanisms, some potentially innocuous such as participants losing interest in the study, others potentially serious such as sicker participants dropping out and some particularly convoluted such as participant death.^8^^,^^9^ Every statistical analysis makes underlying assumptions about how the missing data may influence the estimated results. Conducting examinations on how the missingness might affect study conclusions is vital but often underperformed, often due to unfamiliarity or a lack of readily available statistical code.

We briefly review longitudinal-study missing-data problems and methods to address them; describe a ‘shared-parameter model’ (SPM) approach;^10^^,^^11^ discuss its utility, limitations and extensions; and provide reproducible example data and code for SPM analyses in SAS, Stata and R programming languages.

### Motivating example: cognitive decline and dementia in the Atherosclerosis Risk in Communities (ARIC) study

**Figure 1:**
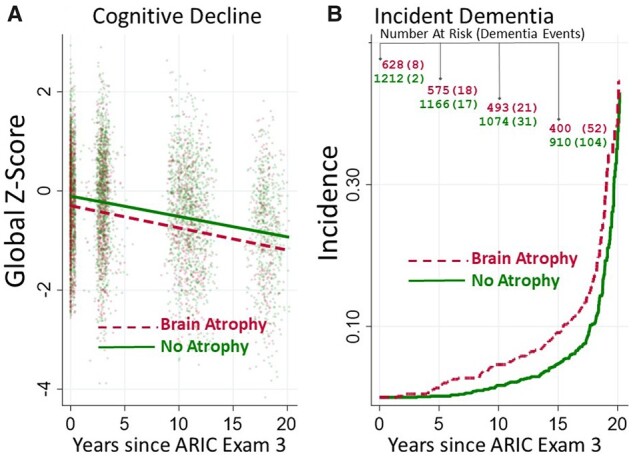
(A) Cognitive decline and (B) incident dementia cases over time by MRI-determined brain-atrophy status in the ARIC study. MRI, magnetic resonance imaging; ARIC, Atherosclerosis Risk in Communities.

We illustrate our approaches using an examination of MRI-defined brain-atrophy associations with 20-year cognitive decline in the ARIC study. Full details on the ARIC study design are published elsewhere.^12^ Briefly, our subset of ARIC participants had brain-atrophy measurements, with atrophy defined as present/absent by brain MRI^13^ at visit 3 (1993–1996) alongside a concurrent battery of cognitive instruments (*N* = 1840; ages 50–73 years, 60% female, 50% Caucasian). Up to four follow-up cognitive assessments occurred over the next 20 years. The longitudinal outcome of interest was a composite cognitive outcome (‘z-score’) derived from the memory, language and executive-function assessments that were performed at each of the visits.^14^ In addition, dementia status was ascertained over time using neuropsychological testing, telephone calls with participants or proxies, hospital dementia code surveillance and death-certificate dementia codes.^15^ The majority (61%) of the original *N* = 1840 did not complete all the follow-up exams, more participants with brain atrophy dropped out (67%) vs participants without atrophy (58%) and those with brain atrophy had higher rates of dementia than those without brain atrophy (Table 1, Figure 1 and Supplementary Tables S1 and S2, available as Supplementary data at *IJE* online). Given these amounts and patterns of missingness, and clear connections between censoring dementia events and missing cognitive-decline outcome data, standard analysis methods may violate the assumptions that underlie their results.

**Table 1:** Characteristics of participants at the index exam (visit 3) by completion status

Characteristic	Total (*N* = 1840)	Completers (*N* = 710) (39%)	Lost to follow-up (*N* = 1130) (61%)	*p*-value
Mean age (SD) (years)	62.84 (4.48)	61.35 (4.10)	63.82 (4.45)	<0.001
Male [*n* (%)]	725 (39%)	254 (36%)	471 (42%)	0.012
Black [*n* (%)]	898 (49%)	342 (48%)	556 (49%)	0.666
Years of education [*n* (%)]				
<High school	477 (26%)	108 (15%)	369 (33%)	<0.001
High-school grad	631 (34%)	256 (36%)	375 (33%)	
>High school	730 (40%)	345 (49%)	385 (34%)	
Smoking status [*n* (%)]				
Current	331 (18%)	97 (14%)	234 (21%)	<0.001
Former	677 (37%)	267 (38%)	410 (36%)	
Never	825 (45%)	344 (49%)	481 (43%)	
Alcohol-drinking status [*n* (%)]				
Current	694 (38%)	314 (44%)	380 (34%)	<0.001
Former	426 (23%)	143 (20%)	283 (25%)	
Never	714 (39%)	251 (35%)	463 (41%)	
Diabetes [*n* (%)]	314 (17%)	79 (11%)	235 (21%)	<0.001
Hypertension [*n* (%)]	874 (48%)	281 (40%)	593 (53%)	<0.001
APOE4 allele [*n* (%)]	594 (32%)	190 (27%)	404 (36%)	<0.001
Mean global z-score (SD)	−0.23 (0.98)	0.03 (0.91)	−0.40 (0.98)	<0.001
Brain atrophy [*N* (%)]	628 (34%)	206 (29%)	422 (37%)	<0.001

### Missing-data categories and related statistical methods

The taxonomy for missing data has been well detailed,^4^^,^^16^^,^^17^ with fairly accepted modelling approaches for each (see Box 1, where missingness assumptions are depicted as occurring over a continuum). Briefly, suppose the full set of data planned to be collected is Y^full^ = (Y^obs^, Y^miss^), where Y^obs^ is actually observed and Y^miss^ is unfortunately missing, with M denoting missingness indicators that separate Y^full^ into its observed and missing components, and the joint distribution of outcomes and missingness being P(Y^full^, M). Under missing completely at random (MCAR): P(M|Y^full^) = P(M) and MAR: P(M|Y^full^) = P(M|Y^obs^), the missing outcome values (Y^miss^) are assumed to be unrelated to M, whereupon missingness would be ignorable. We include the additional description of ‘extended-MAR’ (XMAR)^18–20^ and corresponding partly ignorable missingness assumptions, which are weaker than missing at random (MAR),^21^ where it becomes reasonable to assume that part of the missingness may be ignored by incorporating additional information, denoted by *g*(M, Y^miss^); under XMAR: P{M|Y^full^} = P{M|Y^obs^, *g*(M, Y^miss^)}. The XMAR designation helps to clarify distinctions between standard mixed models, where missingness is ignorable under MAR, the family of ‘conventional’ SPMs described here, which are partly (latently) ignorable under XMAR, and a generalized class of SPMs (GSPMs) that allow further non-ignorable, missing not at random (MNAR) formations.^18–20^ The GSPM uses multiple sets of non-identifiable random effects to allow MNAR structures, whereas the conventional SPM assumes a specific tractable set of random effects as the sufficient information function *g*(M, Y^miss^) (see Supplementary Appendix 1, available as Supplementary data at *IJE* online, for additional details).

**Box 1 Missing-data assumptions and statistical methods**

- MCAR: missing completely at random; completely ignorable missingness; P(M|Y^full^) = P(M)
- Assumption: Missingness is completely random and not related to any observed or unobserved data.
- Example: Unobserved cognitive-decline outcomes occur due to the study ending.
- Analyses: Generalized estimating equations (GEEs),^22^ mixed models.^23^
- Notes: MCAR is a strong assumption, often difficult to justify.
- MAR: missing at random; conditionally ignorable missingness; P(M|Y^full^) = P(M|Y^obs^)
- Assumption: Missingness only depends on observed data and is unrelated to any unobserved data.
- Example: Unobserved cognitive declines among participants dropping out of a study are expected to be similar to the observed declines among participants remaining in the study.
- Analyses: Mixed models,^23^ multiple imputation (MI),^5^ weighting (IPW)^6^ and EM-based.^4^
- Notes: Rich sets of longitudinal outcomes and predictors are often used to support MAR assumptions.
- XMAR: extended missing at random; partly ignorable missingness; P{M|Y^full^} = P{M|Y^obs^, g(M, Y^miss^)}
- Assumption: Missingness depends on observed data and a function of the missing values, g(M, Ymiss).^21^
- Example: Unobserved cognitive declines among participants with a censoring diagnosis of dementia are expected to follow a latent trajectory that is associated with their dementia-censoring time.
- Analyses: SPM (‘conventional-SPM’^18–20^) extended MI.^24^
- Notes: Overall missingness is postulated into ignorable and non-ignorable structures, allowing more tractable handling of potentially remaining informative effects. Additional external data sources containing information related to the outcome and the reason(s) for the outcomes being missing can be essential.
- MNAR: missing not at random; non-ignorable missingness; P(M|Y^full^) = P(M|Y^obs^, Y^miss^)
- Assumption: Missingness depends on the unobserved data (‘informative missingness’).
- Example: Participants drop out of a study because they experience steeper cognitive declines than otherwise similar participants who remain in the study, potentially regardless of dementia ascertainment.
- Analyses: pattern-mixture models (PMMs),^4^^,^^16^ selection models (SEMs)^4^^,^^16^ and generalized shared-parameter models (GSPMs).^19^
- Notes: MNAR situations can induce severe bias; analyses only succeed in eliminating bias under additional unverifiable assumptions, making sensitivity analyses an important strategy.^25^^,^^26^

A primary goal of missingness examinations is to understand effects that assumptions about unobserved outcomes may have on results; generally, this incurs additional computational and modelling costs. Understanding underlying missingness assumptions and being able to efficiently execute modern models are paramount to advancing more robust conclusions. Common analyses used for longitudinal studies include GEEs,^22^ which operate under MCAR assumptions, and mixed models,^23^ which operate under MAR assumptions; both are easy to implement. In the ARIC example, though, participants have no further cognitive assessments after dementia is diagnosed. The *unobserved cognitive data* for the *dementia* participants is likely far *lower* than the *observed cognitive data* for the *non-dementia* participants who remained in the study, even if these participants had otherwise similar observed characteristics (age, sex, baseline cognition, etc.). Basic MCAR or MAR structures are therefore unlikely, whereby standard GEE and mixed-model analyses could yield biased results. We examine an SPM approach in the next section.

## Methods

Three main modelling approaches for informatively missing data have arisen: selection (SEM), pattern-mixture (PMM) and shared-parameter (SPM) models.^16^^,^^20^^,^^27^ SEM and PMM approaches are defined by alternative decompositions of the outcome and missingness processes; both have been detailed extensively, are useful for examining MNAR and have been recommended by statisticians often, but neither is widely used. SPMs can be expressed in either SEM or PMM forms^20^ but, in an SPM, the outcome and missingness processes are both conditioned on latent variables, after which forms of independence are often assumed.^18^^,^^19^ SPMs received less early attention due to both higher computational demands and more uncertainty in influences of the latent assumptions. Clarity in SPM assumptions, generalizations that allow further sensitivity analyses and advances in computation have allowed SPMs to become more attractive. We focus here on SPM methods given their rise in use, flexibility, extendibility and progress in computational approaches.

### The SPM

SPMs extend and connect two of the most widely used statistical models: *mixed models* for longitudinal data and *event/survival models* for event-time data.

*Mixed models*^23^ are commonly used to study trends in repeated outcomes measured over time, such as cognitive decline. As discussed above, standard mixed models inherently make a MAR assumption. Mixed models conceptualize a person as having inherent, underlying, ‘latent characteristics’ that account for the correlation in their repeated measurements. For example, one participant may exhibit ‘worse cognition’ (e.g. a lower baseline cognitive score and faster cognitive decline) than another participant, even when all measured predictors (age, gender, education, etc.) are the same. These underlying, ‘latent cognition’ characteristics are commonly included in the mixed model as person-specific baseline cognitive scores and cognitive declines (random intercepts and slopes).

*Event/survival models*,^28^ such as proportional-hazards models (PHMs), are commonly used to study time-to-event outcomes, such as incident dementia or death. Here, interest is often in factors that predict faster rates of the event occurring (‘hazards’) and event times are only known for those participants who experienced the event. Participants who have not yet experienced an event are ‘censored’ at their last observed follow-up time.

*SPMs*^10^^,^^11^^,^^18–21^^,^^29^ are a type of statistical ‘joint model’,^30^^,^^31^ where information is shared between two or more analysis ‘sub-models’ as shown in Box 2. An SPM uses a mixed model for longitudinal outcomes (e.g. cognition over time) and an event/survival model for an important censoring event directly related to the outcome (e.g. dementia). Information is shared between these longitudinal and event sub-models through a set of latent characteristics (e.g. ‘random effects’). The event sub-model uses parameters called ‘loading factors’ to connect censoring events to the longitudinal sub-model outcomes. When the loading parameters are estimated to be non-zero, there is evidence that the longitudinal and event subprocesses are connected, and the SPM (XMAR) model may be preferred over simpler GEE (MCAR) or GLMM (MAR) models. Whereas the conventional SPM in Box 2 assumes the outcomes and missingness are independent after conditioning on a single set of random effects (an XMAR assumption), the family of Generalized-SPM (GSPM) allows for further MNAR specifications with the inclusion of additional sets of random effects.^18–21^ In our brain-atrophy and cognitive-decline example, dementia represents a potentially egregious source of influential missingness. If the dementia loading factor *ρ*_0_ in Box 2 is estimated as negative in the SPM, this would translate into participants with ‘poorer underlying cognition’ developing dementia faster, leading to additional missing cognitive data (XMAR at a minimum). It is well known that the observed data cannot be used to distinguish MNAR situations from MCAR/MAR/XMAR (since the distinction depends inherently on the data that are missing). However, in some cases, the observed data help to examine whether the inclusion of additional covariates supports MAR assumptions over MCAR. Analogously, one can use the observed data and conventional metrics such as the Bayesian Information Criteria (BIC) to examine whether the SPM inclusion of the additional censoring event sub-models supports potential XMAR assumptions over the simple mixed-model MAR assumptions.^32^ We demonstrate this in our motivating example. Additional technical details on our GEE, mixed and SPM formulations, as well as further explanations of GSPM extensions and partly ignorable structures, are provided in Supplementary Appendix 1, available as Supplementary data at *IJE* online.

**Box 2: Anatomy of a shared-parameter model (SPM)**

- Longitudinal sub-model: (e.g. mixed model)
-
Mixed model:outcome over time=predictors+latent characteristics

-
$\text{Example}:\;E(\text{Cognition})=\beta_{0}+\beta_{1}\text{Time}+\beta_{2}\text{Atrophy}+\beta_{3}\text{Time}\cdot \text{Atrophy}+b_{0i}$

- Event sub-model: (e.g. PHM or other survival model)
-
PHM:  log(Event hazard(t))=baseline hazard+predictors+latent characteristics

-
$\text{Example}:\;\;\text{log}(\text{Dementia hazard(t)})=h_{0}\left(t\right)+\alpha_{1}\text{Atrophy}+\rho_{0}\cdot b_{0i}$

- Shared latent effects:
- Random effects: random intercepts, slopes, etc.
- Example: random intercept, b_0i_ ∼ N(0,τ^2^)
- Principal interpretations:
- β_3_: difference in cognitive decline between those with brain atrophy compared with those without brain atrophy
- b_0i_: subject-specific (latent) random intercept representing a person’s underlying difference in baseline cognition from the baseline of otherwise similar people; often, but not always, assumed to be normally/Gaussian-distributed
- exp(α_1_): dementia hazard ratio associated with brain atrophy
- ρ_0_: loading factor describing association of subject-specific (latent) baseline cognitive scores with dementia hazards
- τ: heterogeneity (standard deviation) of subject-specific (latent) baseline cognitive scores across individuals
- Conventional SPM specification:
- For subjects i = 1..*N*, measured at time points j = 1..*n*_i_, with predictor sets X_ij_, Z_ij_, X*_ij_, Z*_ij_, random-effects vector b_i_, parameter vectors β, α, ρ, and covariance matrix τ:
- Sub-model 1: longitudinal outcome Y_ij_$Y_{\text{ij}}=X_{\text{ij}}\;\beta +{\;Z}_{\text{ij}}b_{i}+\varepsilon_{\text{ij}}$$\varepsilon_{\text{ij}}∼N(0,\sigma^{2})$
- Sub-model 2: event time T_i_ = min($T_{i}^{*}$ ,C_i_), where $T_{i}^{*}$ is the true time to a censoring event with potential information about Y_ij_ and C_i_ is an independent stochastic censoring mechanism. Letting h_0_(t) denote the baseline hazard function and $X_{\text{ij}}^{*}$, $Z_{\text{ij}}^{*}$, being predictor sets potentially different from those in Sub-model 1: $h_{i}\left(t\right)=h0\left(t\right)\cdot \;\text{exp}\;\{X^{*}{}_{\text{ij}}\alpha +\rho Z^{*}{}_{\text{ij}}b_{i}\}$
- Shared random effects: often assumed multivariate-normal (MVN) $b_{i}\sim \;\text{MVN}(0,\tau ).$

## Results

Participants who completed cognitive assessments were more likely to have higher baseline cognitive function, more education and younger age, and were generally in better health (Table 1). Missingness mechanisms are likely at least MAR (not MCAR), since missingness is related to observed covariates. Importantly, brain atrophy was heavily related to completion status; 42% of participants without atrophy completed cognitive assessments vs 33% completing among those with atrophy (Supplementary Table S1, available as Supplementary data at *IJE* online).

Figure 1 shows cognitive trajectories from the observed data (panel A) and Kaplan–Meier functions for censoring dementia events (panel B), stratified by brain-atrophy status. Are the cognitive declines different? Whereas the plotted lines in panel A may appear fairly parallel, they are based on the observed cognitive outcome data (MAR assumption). Since participants with atrophy had lower baseline cognitive scores, higher dementia hazards and more missing data, the plotted MAR lines in panel A may be misleading if XMAR or MNAR missingness is at play.

Table 2 compares estimates of brain-atrophy associations with 20-year cognitive decline across different missingness assumptions. Comparing participants with brain atrophy vs those without, GEE estimates show an unsupported additional cognitive decline of −0.081 standard deviations (SDs) (95% confidence interval: −0.204, 0.042) *p* = 0.199 (under exchangeable correlation; similar for independent, AR1 and unstructured). These estimates are only valid under the strongest assumption (MCAR) which, per above, is highly improbable. The standard mixed-model estimates the additional cognitive decline with brain atrophy to be a bit stronger at −0.111 SD (−0.235, 0.012) *p* = 0.077. The mixed-model estimates are valid under the MAR assumption that, although there may be differences between completers and non-completers, the differences can be explained by what we have observed and is included in the model. However, it is more likely that participants lost to dementia would have had steeper cognitive declines, rather than cognitive declines similar to the observed non-dementia participants as assumed by MAR. The joint SPM under an XMAR assumption estimates a statistically supported additional cognitive decline with brain atrophy of −0.134 SD (−0.257, −0.010) *p* = 0.034, with diagnostic BIC supporting the SPM over the standard mixed model (lower is better). If one considers the SPM XMAR assumptions and results as likely being the closest to the truth (per missingness patterns discussed above), then estimated effect sizes from standard mixed-model (MAR) and GEE (MCAR) estimates are inappropriately attenuated at 17% and 40%, respectively, due to less accurate missingness assumptions. Whereas additional MNAR elements might still remain, if effects are similar to those for dementia, these patterns imply that, as one moves further down the missingness spectrum towards MNAR, brain-atrophy associations with cognitive decline may become stronger.

**Table 2:** Brain-atrophy associations with 20-year cognitive decline across missing-data assumptions from GEE (MCAR), GLMM (MAR) and SPM (extended-MAR: XMAR) models

Model (assumption)	Decline without brain atrophy	Decline with brain atrophy	Additional decline with brain atrophy	Effect attenuation	AIC	BIC
SPM (XMAR)	−0.951, *p* < 0.001	−1.084, *p* < 0.001	−0.134, *p* = 0.034	-ref-	18 477	18 735
	(−1.014, −0.887)	(−1.192, −0.976)	(−0.257, −0.010)			
GLMM (MAR)	−0.918, *p* < 0.001	−1.030, *p* < 0.001	−0.111, *p* = 0.077	17%	18 669	18 900
	(−0.982, −0.855)	(−1.136, −0.924)	(−0.235, 0.012)			
GEE (MCAR)	−0.925, *p* < 0.001	−1.006, *p* < 0.001	−0.081, *p* = 0.199	40%	–	–
	(−0.988, −0.862)	(−1.112, −0.900)	(−0.204, 0.042)			

Adjusted for: age, male, site-race, education and APOE4 status. Decline: estimated absolute 20-year cognitive decline marginalized over adjustors. Longitudinal outcomes were standardized general cognition (z-scores). Cells contain estimates, *p*-values and (95% CIs). SPM, shared-parameter model; GLMM, generalized linear mixed model; GEE, generalized estimating equation; AIC, Akaike Information Criterion (lower is better); BIC, Bayesian Information Criterion (lower is better); AIC/BIC not available for GEE (which is quasi-likelihood-based).

Supplementary Table S3, available as Supplementary data at *IJE* online, additionally provides estimates and percent changes for all parameters in the SPM (XMAR) vs separate MAR models. The SPM results in Supplementary Table S3, available as Supplementary data at *IJE* online, additionally show that the connections (‘loading factors’) between the informative dementia-censoring events and cognitive trajectories were supported statistically. Loading-factor estimates showed that both lower baseline cognition (ρ_0_) HR = 2.57 (1.95, 3.39) per SD and steeper cognitive declines (ρ_1_) HR = 1.08 (1.07, 1.10) per SD were associated with increases in the hazards of dementia-censoring events.

**Figure 2:**
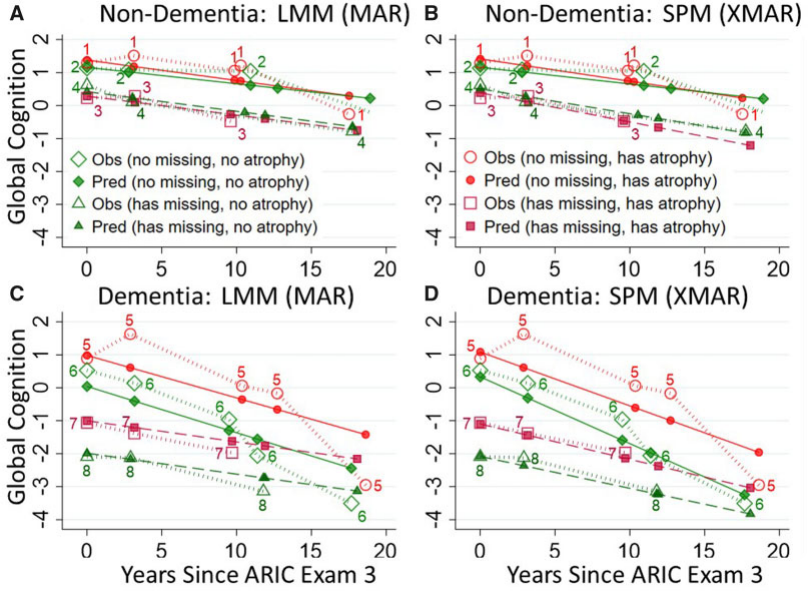
Predicted vs observed values for (A) participants *without dementia* using standard linear mixed-model (LMM) estimates, (B) participants *without dementia* using shared-parameter model (SPM) estimates, (C) participants *with dementia* using standard LMM estimates and (D) participants *with dementia* using SPM estimates. Participants 1 and 5 have complete data and brain atrophy; participants 2 and 6 have complete data and no atrophy; participants 3 and 7 have incomplete data with atrophy; and participants 4 and 8 have incomplete data without atrophy. LMM, linear mixed model; SPM, shared-parameter model; MAR, missing at random; XMAR, extended missing at random.

Figure 2 shows the observed and predicted values for eight participants with different atrophy, dementia and missingness status. Panels A and B show non-dementia participants 1 (without atrophy) and 2 (with atrophy); both have complete data and similar predicted values comparing mixed-model results to SPM. Non-dementia participants 3 (without atrophy) and 4 (with), both having missing data, show SPM predictions closer to their observed values than the mixed model. Panels C and D show similarly structured participants with dementia, three of whom (participants 6, 7 and 8) all show closer fits to their observed data with the SPM. The fourth (participant 5) with complete data has similar mixed and SPM predictions. These eight participants are indicative of overall results, where correlations between SPM predictions and observed values were slightly higher with SPM (Pearson’s = 0.954) vs mixed models (Pearson’s = 0.945), with improvements in all eight atrophy by dementia by completeness groups and the largest improvements being in those with incomplete data (Supplementary Figure S1, available as Supplementary data at *IJE* online). Here, even when considering observed data metrics (AIC/BIC, loading-factor 95% CIs, observed vs predicted correlations), the XMAR assumptions of the SPM are supported over the MAR assumptions of the standard mixed model.

### Simulated data and reproducible code to run SPMs

To facilitate the broader implementation of SPMs, simulated data based on the ARIC cognitive-decline example and reproducible programs are provided in the Supplementary Materials, available as Supplementary data at *IJE* online, and at github link https://github.com/MichaelGriswold/SPM. The simulation approach and data are found in Supplementary Appendix 2, available as Supplementary data at *IJE* online. General ‘pseudo-code’ and tips to help convergence are provided (Supplementary Appendix 3, available as Supplementary data at *IJE* online), along with specific code for Stata, SAS and R programming languages (Supplementary Appendices 4–6, available as Supplementary data at *IJE* online) and corresponding results (Supplementary Tables S5–S7, available as Supplementary data at *IJE* online). Others have used simulations to prove statistical properties of SPMs; our simulated data are meant only to provide a starting place for code implementation.

## Discussion

Missing data in longitudinal studies can significantly bias study results. Many techniques exist to explore the degree of this potential bias, but routine implementation has been slow, despite guidelines recommending inclusion. We have described how an underutilized method, i.e. the SPM, can be used to examine potentially important missing-data effects, using a real-world example to demonstrate the approach. We have additionally provided reproducible data and code to facilitate and broaden SPM use. In accordance with other recommendations, we suggest the sensitivity examinations to be included routinely at least in Supplementary Materials, available as Supplementary data at *IJE* online. When sensitivity analyses suggest that conclusions may depend on missing-data assumptions, discussion of how primary results may be affected by important missingness elements is prudent.

Another feature of joint models is their flexibility for extensions. Here, we have focused on concepts to outline a simple, conventional SPM specification (see Supplementary Appendix 1, available as Supplementary data at *IJE* online, for additional technical details). Extensions are vast and include useful aspects such as: adding multiple longitudinal sub-models (e.g. simultaneously modelling declines in language, memory and executive function, or even multiple individual cognitive instruments, similar to factor analysis or structural equation modelling); adding multiple-event sub-models as competing risks (e.g. times to dementia, nursing-home institutionalization, death); sharing time-dependent predictions from the longitudinal sub-models (vs only random effects); including flexible random-effect distributions (such as mixture models), including additional random effects for MNAR examination, and many other augmentations. The broader class of joint models is an active area of statistical research.

Deciding how to deal with deaths deserves special attention in any longitudinal data analysis.^8^^,^^9^^,^^16^^,^^33^ Mixed models implicitly borrow information from the observed data and attribute it to the missing data. This property extends the missing-data assumptions from MCAR to MAR but does not distinguish between missing data for those who remain alive vs for those who have died. Others have described mixed models as conveying inferences on an ‘immortal cohort’. We used a simple SPM with a single longitudinal outcome (global cognition) and a single censoring event (dementia) in order to describe and illustrate key SPM aspects. When we extended the SPM to incorporate competing risk sub-models for both dementia and death events, associations between brain atrophy and cognitive decline were even stronger and supported by BIC and loading parameter estimates similar to those described above (see Supplementary Appendix 7, available as Supplementary data at *IJE* online, for description and results). Additional GSPM formulations with assumptions that admit ‘non-future dependence’^19^ could be used to explore death effects further. Accounting for death effects is challenging by any method, particularly when informative missingness is involved, and, although this has been an active area of statistical research, a broader platform of ‘mortal-inference’ methods that assess sensitivity to full MNAR would be valuable.

We follow trends in stating that the conventional SPM can be conceptualized as an ‘extended-MAR’ (XMAR) assumption where, *given the random effects*, missingness is assumed to no longer depend on the unobserved longitudinal measurements or future times (events or censoring).^19^ Under this partly ignorable assumption, a conventional SPM can constitute a valuable missingness examination as shown in our example. However, we note that the partly ignorable assumption is still an assumption and, although it is more flexible than MAR, even more insidious MNAR situations not addressed by a conventional SPM can exist, where differences between observed and missing observations are uninformed by the observed data. Such situations can only be addressed in an extended sensitivity analysis, where one elicits a scientifically plausible range for differences in observed vs missing values and then examines how findings vary within this plausible range.^19^^,^^25^^,^^30^ Recent work for SPM approaches is based on ‘grounding’ the sensitivity analyses on the conventional SPM that we have detailed.^26^ Such analyses are more complex than those illustrated in the present work and could indicate additional sensitivities not identified here. However, assuming that additional informative missingness effects would act in the same direction as dementia (such as those shown by the additional death model, Supplementary Appendix 7, available as Supplementary data at *IJE* online), the stated SPM results here would be conservative if additional effects above and beyond those accounted for in the SPM remain.

## Conclusion

Results from standard GEE (MCAR) and mixed models (MAR) can be biased if the missing-data assumptions are incorrect. It is difficult or impossible to tell whether an MNAR situation is correct because the data needed to test the assumption are, by definition, *missing*. However, just as observed data may support an MAR assumption over an MCAR one, an XMAR assumption may extend the utility of other observed data related to the missing outcomes to show utility over MAR. Recent national recommendations regarding missing-data state: ‘Examining sensitivity to the assumptions about the missing data mechanism should be a mandatory component of reporting.’^2^ Sensitivity analyses should examine scientifically plausible situations with appropriate approaches rather than using single imputation strategies that are known to bias both estimates and standard errors such as best-case, worst-case or last-observation-carried-forward (LOCF).^2^^,^^25^ Our suggested approach is to (i) identify scientifically plausible informative censoring events (like dementia for cognitive decline), then (ii) incorporate these events in an SPM alongside a longitudinal mixed model, (iii) compare the SPM (XMAR) results to estimates obtained from separate GEE (MCAR) and mixed models (MAR), and finally (iv) consider additional MNAR structures that may still remain using GSPM, SEM or PMM methods. This approach uses multiple models, which are all now becoming more and more computationally tractable, to provide a better understanding of potential mechanisms and influences of missing data. To place an addendum on the famous quote by George Box: ‘All models are wrong, but some models are useful’—and many useful models are illuminating.

## Supplementary data

Supplementary data are available at *IJE* online.

## Ethics approval

The institutional review board at each centre approved the study and all participants provided written informed consent at each visit.

## Funding

This work was supported by the National Institutes of Health [HHSN268201100005C, HHSN268201100006C, HHSN268201100007C, HHSN268201100008C, HHSN268201100009C, HHSN268201100010C, HHSN268201100011C, HHSN268201100012C, 2U01HL096812, 2U01HL096814, 2U01HL096899, 2U01HL096902, 2U01HL096917 and R01-HL70825].

## Data availability

Study protocols and data for the ARIC and ARIC Brain MRI Study may be obtained by approved persons through written agreements with the ARIC Steering Committee and the research sponsor (National Institutes of Health, National Heart, Lung, and Blood Institute) (e-mail: tmosley@umc.edu). Reproducible Research example data sets are available at https://github.com/MichaelGriswold/SPM or via e-mail from Dr Griswold (mgriswold@umc.edu).

## Supplementary Material

dyab086_Supplementary_Data
