# Supplementary material for: Reflection on modern methods: shared-parameter models for longitudinal studies with missing data
Source: Int J Epidemiol. 2021 Jun 11;50(4):1384–93. doi: 10.1093/ije/dyab086 (PMC8407871; doi:10.1093/ije/dyab086)
Supplement: dyab086_Supplementary_Data [file dyab086_supplementary_data.zip › ije-2020-03-0395-File006.docx]

**Supplementary Materials**

SPM reproducible research materials are also available at <https://github.com/MichaelGriswold/SPM>

Appendix list with Tables and Figure descriptions:

**Appendix 1: Technical Details**

Text 1.1: Technical specification of primary models used on ARIC example data

Text 1.2: Conventional SPM and Generalized SPM (GSPM) specifications

Text 1.3: Definition of Partly Ignorable, SPM latent ignorability and the missingness continuum

Table S1: Completion and Dementia Rates within Brain Lesion Groups

Table S2: Baseline Characteristics of Completers vs Dementia vs LTFU participants

Table S3: Comparisons of all MAR and XMAR parameter estimates for all SPM submodels

Figure S1: Comparisons of observed values versus predicted values for linear mixed model (LMM) and shared parameter model (SPM) approaches

**Appendix 2: Simulated Data**

Text: Simulation Approach

Table S4: Simulated SPM Data (N=500) - included in separate document

**Appendix 3: SPM Pseudo-code**

Text: Fitting algorithms and convergence tricks

**Appendix 4: Stata Code for SPM**

Table S5: Robust SE Results using Stata code on simulated data

**Appendix 5: SAS Code for SPM**

Table S6: Robust SE Results using SAS code on simulated data

**Appendix 6: R Code for SPM**

Table S7: Model Based SE Results using R code on simulated data

**Appendix 7: SPM with Competing Risks of Dementia and Death (crSPM)**

Text: Technical specification of competing risk model & related code

Table S8: Baseline Characteristics of Completers vs Dementia vs Deaths vs LTFU participants

Table S9: Comparisons of all MAR and XMAR parameter estimates for all crSPM submodels

**APPENDIX 1: TECHNICAL DETAILS**

***1.1: Technical specification of primary models used on ARIC example data***

Overarching Modeling Information:

*Outcomes, predictors and adjustors*: Our longitudinal data analyses examine relationships between cognitive function outcomes (GlobalZ: a continuous composite cognitive “Z-score” measure) over time (yrs: years since baseline) and the primary predictor of an indicator for brain atrophy (atrophy). Covariates used for adjustment (adj) included an indicator for male sex, baseline age (i.e. age at Visit 3) in years, educational attainment (educ: 3-levels; basic, intermediate, and advanced), and a combined race/study-site categorical variable (race-site: 3-levels; Forsyth-White, Forsyth-Black, and Jackson-Black) to account for site-race aliasing.

*Mean Model Specification*: Denoting participant (i) at time (j), all approaches used the following marginal mean model specification for cognitive trajectories over time:

E(GlobalZ*_ij_*) = Xβ*_ij_* = β_0_ + β_1_(years) + β_2_(atrophy) + **β_3_**(atrophy)⋅(years) + β⋅(adj)

Where:

- β_0_ (intercept): Expected cognitive function at the index visit (years=0)
- β_1_: Expected cognitive decline per year for those without atrophy
- β_2_: Difference in expected cognitive function for those with atrophy versus without at baseline
- **β_3_: Difference in expected cognitive decline per year for those with vs without atrophy**

and β⋅(adj) represents the vector of additional regression parameters and design matrix corresponding to the adjustment covariates specified above. Here, the interaction term **β_3_** represents the estimate of primary interest, differences in cognitive decline over time between brain atrophy groups. For the example we use simple linear declines and their difference for exposition purposes, in general we could model more sophisticated potential nonlinear declines by using additional regression terms and parameters. Below we use the common short-hand notation (Xβ*_ij_*) for our mean model.

*Robust Standard Errors:* We used Huber-White robust standard error estimates throughout our results, tables and figures. We also compared model-based (non-robust) estimates which gave similar results for the example (not shown). We generally recommend using robust standard error estimates whenever appropriate but recognize that this option is not yet available in all statistical packages and can occasionally cause convergence issues.

GEE (MCAR) model specification:

Our primary GEE used an exchangeable working correlation structure.

1. GlobalZ*_ij_* = Xβ*_ij_* + e*_ij_*
2. e*_ij_* ~ N(0, σ^2^)
3. Corr(e*_ij_*, e*_ik_*) = ρ (exchangeable)

Robust standard errors were employed. We additionally fit working correlation structures including independent, autoregressive and unstructured which gave similar results and conclusions.

GLMM (MAR) model specification:

Random intercepts, slopes and their covariance were incorporated in our primary mixed model variance structure.

1. GlobalZ*_ij_*|**b***_i_* = Xβ*_ij_* + b*_0i_* + b*_1i_*⋅(years) + e*_ij_*
2. b*_oi_* ~ N(0, τ*_0_*^2^); b*_1i_* ~ N(0, τ*_1_*^2^); Cov(b*_oi_*,b*_1i_*) = τ*_01_*
3. e*_ij_*|**b***_i_* ~ N(0, σ^2^)

Robust standard errors were employed.

Conventional SPM (XMAR) model specification:

During follow-up, measurements on each participant may be lost due to Dementia or right censoring. Let *T_i_* = (*T_i_*, *K_i_*) be the times to Dementia, or censoring on subject *i*, with *K_i_* taking values {0=censoring, 1=dementia }, *T_ki_* = (*T_i_*, *K_i_*=k) indicate the censoring time is due to the *k*th reason, and H(t_1_) be the hazard of dementia at time T_1_=t_1_. Throughout, right censoring is assumed independent of dementia and longitudinal outcomes are assumed independent of the censoring events after conditioning on the shared random effects. The conventional SPM incorporates and extends the GLMM above by specifying:

Longitudinal GLMM submodel

1. GlobalZ*_ij_*|**b***_i_* = Xβ*_ij_* + b*_0i_* + b*_1i_*⋅(years) + e*_ij_*
2. b*_oi_* ~ N(0, τ*_0_*^2^); b*_1i_* ~ N(0, τ*_1_*^2^); Cov(b*_oi_*,b*_1i_*) = τ*_01_*
3. e*_ij_*|**b***_i_* ~ N(0, σ^2^)

Dementia Event Submodel:

1. log{h(T_1_*_i_*|**b***_i_*)} = log{h_0_(T_1_*_i_*)} + α_1_(atrophy) + **α**⋅(**adj**) + ρ_0_b_0i_ + ρ_1_b_1i_
2. T_1_*_i_*|**b***_i_* ~ Weibull

where h_0_(⋅) is the Weibull baseline hazard and **α**⋅(**adj**) represents vectors of additional regression parameters and the design matrix corresponding to the adjustment covariates specified above. Robust standard errors were employed.

***1.2: Conventional SPM and Generalized SPM (GSPM) specifications***

The generalized shared-parameter modeling (GSPM) framework of Creemers (2011) details the family of generalized shared parameter models (GSPM) and subfamily of conventional SPMs as follows:

For subjects *i*=1..N, measured at timepoints *j*=1..n*_i_*, with planned outcomes to be collected ${\boldsymbol{Y}_{\boldsymbol{ij}}^{\boldsymbol{full}}\boldsymbol{=Y}}_{\boldsymbol{ij}}$ , and observation indicators $\boldsymbol{R}_{\boldsymbol{ij}}$ equal to 1 if $\boldsymbol{Y}_{\boldsymbol{ij}}$ is observed and 0 if missing, we have outcome and observation vectors $\boldsymbol{Y}_{\boldsymbol{i}}$and $\boldsymbol{R}_{\boldsymbol{i}}$for the *i*^th^ subject, respectively. Defining parameters ***θ*** for the outcome process and ***ψ***  for the missingness process we can write the full data density as $f(\boldsymbol{y}_{\boldsymbol{i}}\boldsymbol{,r}_{\boldsymbol{i}}\left| \boldsymbol{,} \right)$*.*

Similar to Little (1995), Creemers (2011) next considers augmenting the joint density above using a set of random effects ***b****_i_*, with distributional parameters ***ξ*** : $\boldsymbol{f(}\boldsymbol{y}_{\boldsymbol{i}}\boldsymbol{,r}_{\boldsymbol{i}}\boldsymbol{,}\boldsymbol{b}_{\boldsymbol{i}} \left| \boldsymbol{,}\boldsymbol{,} \right)$*.* Either selection model (SEM) or pattern mixture model (PMM) factorizations can then be used. Under SEM, one obtains:

$$\boldsymbol{f}\left( \boldsymbol{y}_{\boldsymbol{i}}\boldsymbol{,r}_{\boldsymbol{i}}\boldsymbol{,}\boldsymbol{b}_{\boldsymbol{i}} \left| \boldsymbol{,}\boldsymbol{,} \right)\boldsymbol{=f}\left( \boldsymbol{y}_{\boldsymbol{i}} \right|\boldsymbol{b}_{\boldsymbol{i}}\boldsymbol{,} \right)\boldsymbol{f(}\boldsymbol{r}_{\boldsymbol{i}} \left| \boldsymbol{y}_{\boldsymbol{i}}\boldsymbol{,}\boldsymbol{b}_{\boldsymbol{i}}\boldsymbol{,} \right)\boldsymbol{f}\left( \boldsymbol{b}_{\boldsymbol{i}} \right| \boldsymbol{)}$$

The conventional SPM makes the simplifying assumption that the outcome ($\boldsymbol{Y}_{\boldsymbol{i}}$) and missingness ($\boldsymbol{R}_{\boldsymbol{i}}$) processes are independent, conditional upon the random effects (***b****_i_*):

$$\boldsymbol{f}\left( \boldsymbol{y}_{\boldsymbol{i}}\boldsymbol{,r}_{\boldsymbol{i}}\boldsymbol{,}\boldsymbol{b}_{\boldsymbol{i}} \left| \boldsymbol{,}\boldsymbol{,} \right)\boldsymbol{=f}\left( \boldsymbol{y}_{\boldsymbol{i}} \right| \boldsymbol{b}_{\boldsymbol{i}}\boldsymbol{,} \right)\boldsymbol{f(}\boldsymbol{r}_{\boldsymbol{i}} \left| \boldsymbol{b}_{\boldsymbol{i}}\boldsymbol{,} \right)\boldsymbol{f}\left( \boldsymbol{b}_{\boldsymbol{i}} \right| \boldsymbol{)}$$

Creemers (2011) and Njagi (2014) show that this conventional SPM does not meet MAR conditions, and call it instead an extended-MAR assumption to distinguish it from a full MNAR approach, which can be accommodated by the generalized SPM (GSPM) family that Creemers (2011) defines by expanding the sets of random effects used. Partitioning the full set of planned data to be collected $\boldsymbol{Y}_{\boldsymbol{i}}$into its’ observed ($\boldsymbol{Y}_{\boldsymbol{i}}^{\boldsymbol{obs}}$) and missing ($\boldsymbol{Y}_{\boldsymbol{i}}^{\boldsymbol{miss}}$) components, a generalized SPM (GSPM) takes the form:

$$\boldsymbol{f(}\boldsymbol{y}_{\boldsymbol{i}}^{\boldsymbol{obs}}\left| \boldsymbol{g}_{\boldsymbol{i}}\boldsymbol{,}\boldsymbol{h}_{\boldsymbol{i}}\boldsymbol{,}\boldsymbol{j}_{\boldsymbol{i}}\boldsymbol{,}\boldsymbol{l}_{\boldsymbol{i}} \right)\boldsymbol{f(}\boldsymbol{y}_{\boldsymbol{i}}^{\boldsymbol{miss}}\left| \boldsymbol{y}_{\boldsymbol{i}}^{\boldsymbol{obs}}\boldsymbol{,}\boldsymbol{g}_{\boldsymbol{i}}\boldsymbol{,}\boldsymbol{h}_{\boldsymbol{i}}\boldsymbol{,}\boldsymbol{k}_{\boldsymbol{i}}\boldsymbol{,}\boldsymbol{m}_{\boldsymbol{i}} \right)\boldsymbol{f(}\boldsymbol{r}_{\boldsymbol{i}} \left| \boldsymbol{g}_{\boldsymbol{i}}\boldsymbol{,}\boldsymbol{j}_{\boldsymbol{i}}\boldsymbol{,}\boldsymbol{k}_{\boldsymbol{i}}\boldsymbol{,}\boldsymbol{q}_{\boldsymbol{i}} \right)$$

Where $\boldsymbol{g}_{\boldsymbol{i}}\boldsymbol{,}\boldsymbol{h}_{\boldsymbol{i}}$*,*$\boldsymbol{j}_{\boldsymbol{i}}\boldsymbol{,}\boldsymbol{k}_{\boldsymbol{i}}\boldsymbol{,}\boldsymbol{l}_{\boldsymbol{i}}\boldsymbol{,}\boldsymbol{m}_{\boldsymbol{i}}$ and $\boldsymbol{q}_{\boldsymbol{i}}$are independent vectors of random effects and parameters are suppressed for notational convenience. Creemers (2011) states that this is the most general random effects model that one can consider, with $\boldsymbol{g}_{\boldsymbol{i}}$ being shared between all 3 terms, $\boldsymbol{h}_{\boldsymbol{i}}\boldsymbol{,}\boldsymbol{j}_{\boldsymbol{i}}$and$\boldsymbol{k}_{\boldsymbol{i}}$being shared only between pairs of terms, and $\boldsymbol{l}_{\boldsymbol{i}}\boldsymbol{,}\boldsymbol{m}_{\boldsymbol{i}}$ and $\boldsymbol{q}_{\boldsymbol{i}}$ being restricted to inclusion in a single term. In any given application, assumptions are then made on which terms will be included, which then translate into different characterizations of how outcomes and missingness are allowed to relate. For example, they show that assuming that the sets $\boldsymbol{g}_{\boldsymbol{i}}\boldsymbol{,}\boldsymbol{h}_{\boldsymbol{i}}$ and $\boldsymbol{k}_{\boldsymbol{i}}$ are zero, leads to an MAR missingness model under both SEM and PMM factorizations. They go on to define conditions and a sub-class of the GSPM where MAR holds. Using this framework, Creemers (2011) and Njagi (2014) both show that the conventional SPM, where a single, common underlying random-effect structure is assumed (e.g. a subfamily of the above GSPM that only uses $\boldsymbol{g}_{\boldsymbol{i}}$), does not admit MAR. In our work we follow Creemers (2011), who calls the conventional SPM an “extended-MAR” assumption (we denote this XMAR), to distinguish it from other potential assumptions one could make about the remaining sets of GSPM random effects which would lead to other potential MNAR characterizations. Njagi (2014) does not necessarily make this distinction, simply stating that since the narrow (conventional) SPM formulation ‘defies MAR’, that it is MNAR by construction. Our position is that missingness assumptions are more of a journey than a destination, and that the extended-MAR (XMAR) notation appropriately positions a conventional SPM as being between fully ignorable MAR and fully non-ignorable MNAR (see also the related SPM partly ignorable XMAR characterization below). Given that there are many potential random effects one may consider for inclusion, even in the more narrowly defined conventional SPM (random intercepts, slopes, splines, interactions with covariates, etc.), the examination of effects along the spectrum of missingness using SPM and GSPM families is encouraged. Su (2019) gives an example of such a sensitivity analysis.

***1.3: Definition of Partly Ignorable, SPM latent ignorability and the missingness continuum***

Harel and Schafer (2009) detail two ways to formalize the assumption that the missingness process is partly-ignorable: latent ignorability and partial ignorability. They state that, under conditions weaker than MAR, it becomes reasonable to ignore part of the missingness information using coarsened summaries of either the missing values (latent ignorability) or the missing data indicators (partial ignorability). Denoting the full data as Y^full^ = (Y^obs^, Y^miss^), and missingness process indicators as *M*, the joint distribution of outcomes and missingness is:

*P*(Y^full^*,* *M* | ***θ****,* φ) = *P*(Y^full^ | ***θ*** *) P*(*M* | Y^full^*,* φ)

*Latent Ignorability:*

Letting *h*(Y^miss^) denote a coarsened summary of the missing values that were intended to have been collected but were not, missing data are said to be “latently” missing at random given *g*(Y^miss^) if:

*P*(*M* | Y^full^*,* φ) = *P*(*M* | Y^obs^*,* Y^miss^ *,* φ) = *P*{*M* | Y^obs^*, h*(Y^miss^ )*,* φ}

for all possible φ, with *M* and Y^obs^ fixed at their realized values. Using this, Harel and Schafer (2009) show that the conventional SPM family is partly-ignorable via this latent ignorability assumption. In example 7 of the latent ignorability concept they describe that taking the set of random effects shared between the outcome and censoring event processes as the coarsened summary, *h*(Y^miss^) = ***b****_i_*, leads to latent ignorability. Hence, the conventional SPM operates under a partly-ignorable assumption (latent ignorability), characterizing it as extended-MAR (XMAR).

*Partial Ignorability:*

Letting *g*(*M*) denote a coarsened summary of the set of missing-data indicators *M,* φ =(γ, δ), and factoring the missingness process as:

*P*(*M* | Y^full^*,* φ) = *P*{g(*M*) | Y^full^*,* γ)} *P*{*M* | Y^full^*,* g(*M*), δ)}

Missing data are said to be “partially” missing at random given *g*(*M*) if:

*P*{*M* | Y^full^*,* g(*M*), δ)} = *P*{*M* | Y^obs^*,* g(*M*), δ)}

for all possible δ, with *M* and Y^obs^ fixed at their realized values. Harel and Schafer (2009) show that partial ignorability is a useful extended-MAR (XMAR) concept for a variety of examples, including factoring the overall missingness mechanism into two parts, one that partitions Y^full^ = (Y^obs^, Y^miss^) and one that partitions the missing values into two separate processes Y^miss^ = (Y_1_^miss^, Y_2_^miss^).

*Partly-Ignorable:*

Putting Harel and Schafer’s (2009) separate definitions for specifying that part of the missing data mechanism is ignorable (latent/partial ignorability) together for convenience, we have that missing data are said to be “partly” missing at random given *g*(M,Y^miss^) = *h*(Y^miss^) if latent ignorability holds, or *g*(M,Y^miss^) = *g*(M) if partial ignorability holds. Positioning extended-mar (XMAR) and the partly-ignorable assumption in the standard taxonomy of missingness, we have:

**MCAR**: P(M | *Y*_com_) = P(M) - completely ignorable

< **MAR**: P(M | *Y*_com_) = P(M | *Y*_obs_) - ignorable

< **XMAR**: P(M | *Y*_com_) = P(M | *Y*_obs_, *g*(M,Y^miss^)) - partly-ignorable (latent/partial)

< **MNAR**: P(M | *Y*_com_) = P(M | *Y*_obs_, *Y*_mis_) - non-ignorable


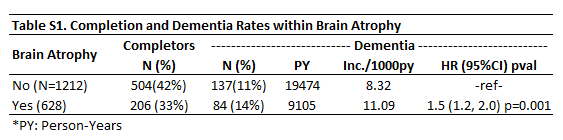


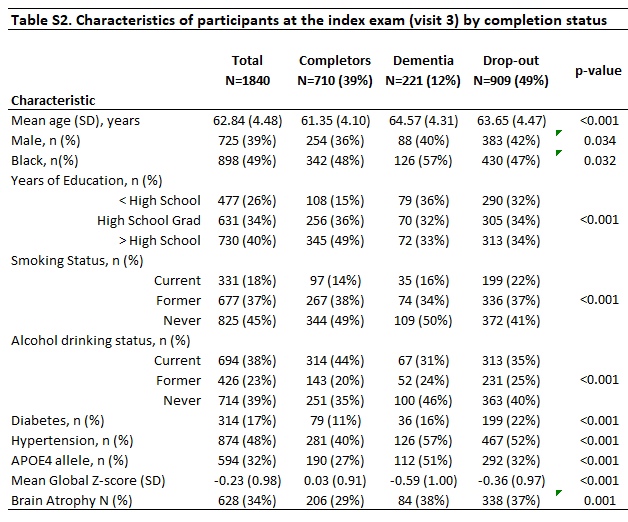


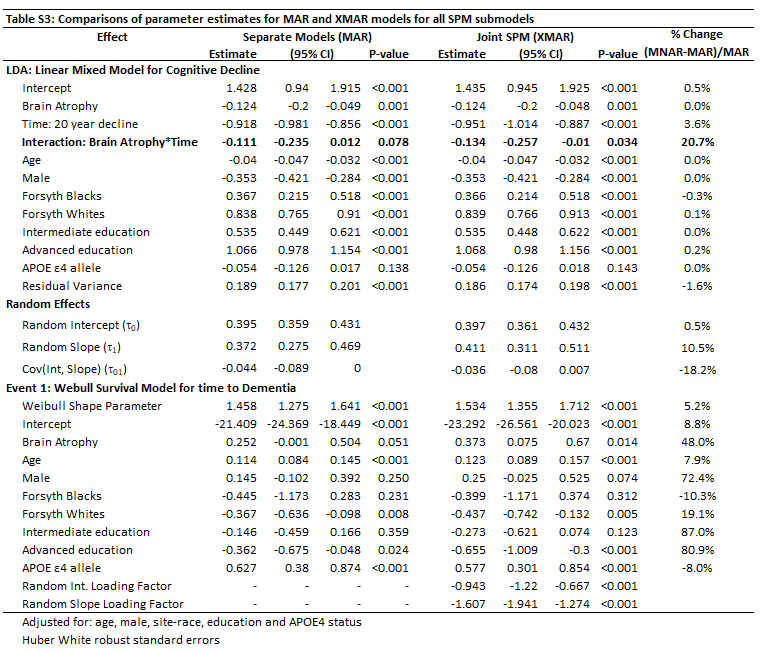


**Figure S1:** Comparisons of observed values versus predicted values for linear mixed model (LMM) and shared parameter model (SPM) approaches; Scatterplot matrix (Panel A) and Pearson correlations by atrophy, dementia, and completeness groupings (Panel B).


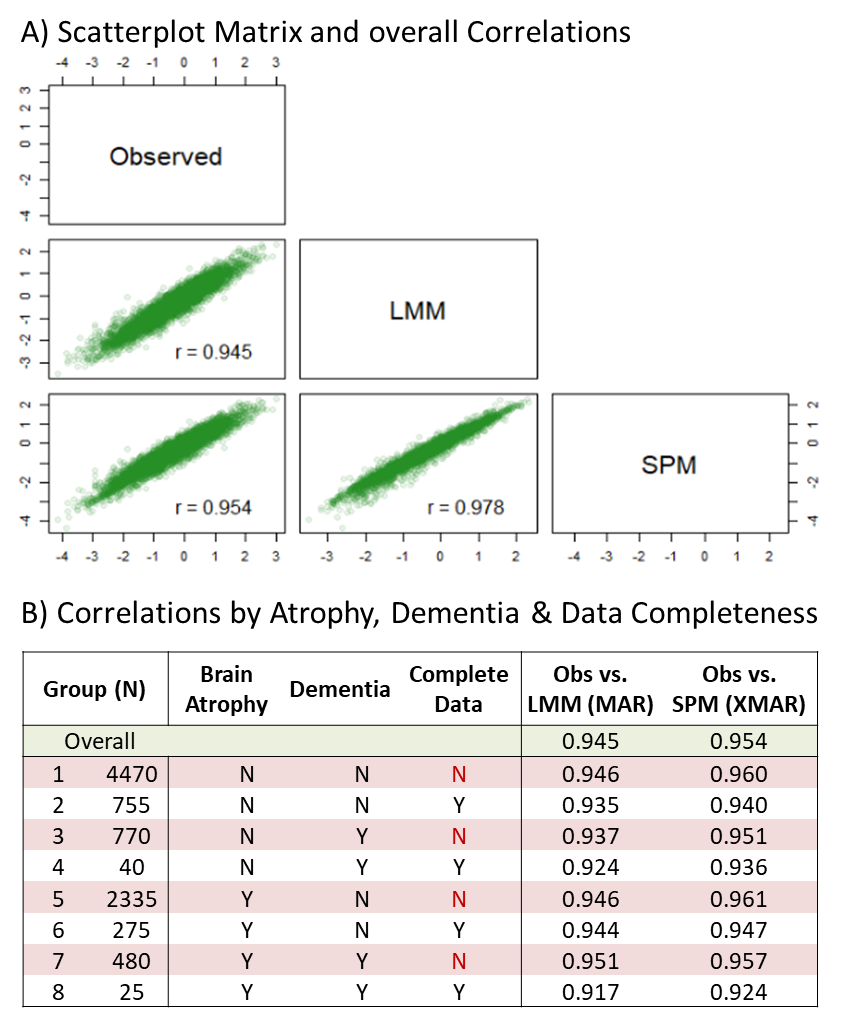


**APPENDIX 2: SIMULATED DATA**

To provide reproducible code and results for others to use we generated simulated data with characteristics similar to our ARIC motivating example data and comparable to other standard longitudinal cohort study datasets. For simplicity, we chose to simulate N=500 participants having a maximum of 4 visits each, with brain atrophy modifying linear cognitive decline trajectories and sex and age as adjustors. Our simulation steps to generate N=500 participants were as follows (created variables are italicized) and the resultant data may be found in Supplementary Table S4:

Step 1: Use the ARIC data to obtain simulation parameters:

1. Using the ARIC data we fit the brain atrophy linear-trajectories mixed model:
   1. E(GlobalZ*_ij_*) = Xβ*_ij_* = β_0_ + β_1_years + β_2_atrophy + **β_3_**(atrophy)⋅(years) + β_8_male+ β_9_age
   2. GlobalZ*_ij_*|**b***_i_* = Xβ*_ij_* + b*_0i_* + b*_1i_* years + e*_ij_*
   3. b*_oi_* ~ N(0, τ*_0_*^2^) ⊥ b*_1i_* ~ N(0, τ*_1_*^2^)
   4. e*_ij_*|**b***_i_* ~ N(0, σ^2^)
2. Using the ARIC data we fit a Weibull survival model for Dementia times
   1. log{H(T_1_*_i_*)} = log{H_0_(T_1_*_i_*)} + α_1_atrophy + α_2_male+ α_3_age
   2. T_1_*_i_* ~ Weibull(a_1_)
3. We then saved all regression parameter, variance component estimates and Weibull shape parameters (**β**, τ*_0_*, τ*_1_*, σ, **α**, a_1_) for use in simulating new data.

Step 2: Simulate the design matrix (predictor variables) for N=500 mock participants

1. Generate N=500 *id* numbers with *visit* = 1,2,3,4 for each (2000 total observations)
2. Generate a *years* from baseline variable with distribution similar to observed ARIC years
   1. If *visit*==1 then *years* = 0
   2. If *visit*==2 then *years* ~ Normal(3, 0.5)
   3. If *visit*==3 then *years* ~ Normal(10, 1)
   4. If *visit*==4 then *years* ~ Normal(18, 1)
3. Generate a *male* indicator variable for each id with probability = 40%
4. Generate a continuous baseline age variable for each id as *age* ~ Normal(65, 5)
5. Generate an *atrophy* indicator variable for each id with probability = 34%
6. Generate random intercepts for each id as *b_oi_* ~ N(0, τ*_0_*^2^) with τ*_0_* from ARIC estimates in Step 1
7. Generate random slopes for each id as *b_1i_* ~ N(0, τ*_1_*^2^) with τ*_1_* from ARIC estimates in Step 1

Step 3: Simulate longitudinal and survival outcomes under an approximate SPM framework:

1. Simulate latent cognition effects (random intercepts and slopes) for each id:
   1. b*_oi_* ~ N(0, τ*_0_*^2^) ⊥ b*_1i_* ~ N(0, τ*_1_*^2^)

1. Simulate complete cognitive outcome trajectories for each id:
   1. Calculate fixed-effect linear predictors Xβ*_ij_* using the regression parameters from Step 1 and the generated covariates in Step 2.
   2. Generate residual errors e*_ij_* ~ N(0, σ^2^) with σ from ARIC estimates in Step 1
   3. Generate cognitive outcome variables for each timepoint:
      1. *GlobalZ_ij_*|**b***_i_* = Xβ*_ij_* + b*_0i_* + b*_1i_*⋅years + e*_ij_*

1. Simulate Dementia times for each id:
   1. Generate Dementia times as T_1_*_i_* ~ Weibull(a_1_, b_1_ = exp(**Xα_1_** + ρ_10_b_0i_ + ρ_11_b_1i_)^(-1/a_1_)) using the Weibull shape and regression parameters from ARIC estimates in Step 1
2. Simulate Censoring times for each id:
   1. Generate Administrative Censoring times as T_0_*_i_* = min(C_1_*_i_*, C_2_*_i_*) where
      1. C_1_*_i_* ~ Weibull(a_1_, b_1_)
      2. C_2_*_i_* ~ Normal(17, 1)
3. Calculate the Dementia or Censoring time for each id:
   1. Calculate *T_i_* = min(T_0_*_i_*, T_1_*_i_*) and set *K_i_*=0, 1 corresponding to the minimum time being Censoring or Dementia respectively.
4. Induce missingness in the longitudinal cognitive outcome data corresponding to the coarsening times *T_i_*  by dropping visits where *T_i_*  < years*_i_* for each id (e.g. remove outcome data after dementia or censoring events were simulated to occur)

The resultant dataset is given in Supplementary Table S4 (first 7 subjects copied below).

| id | visit | years | brainloss | age0 | male | demyears | dementia | globz | globz_complete |
| --- | --- | --- | --- | --- | --- | --- | --- | --- | --- |
| 1 | 1 | 0.00 | 0 | 62.39 | 0 | 25.66 | 0 | -0.63 | -0.63 |
| 1 | 2 | 3.52 | 0 | 62.39 | 0 | 25.66 | 0 | -0.61 | -0.61 |
| 1 | 3 | 10.60 | 0 | 62.39 | 0 | 25.66 | 0 | -0.36 | -0.36 |
| 1 | 4 | 16.89 | 0 | 62.39 | 0 | 25.66 | 0 | -0.68 | -0.68 |
| 2 | 1 | 0.00 | 1 | 63.94 | 1 | 24.01 | 0 | -1.49 | -1.49 |
| 2 | 2 | 3.28 | 1 | 63.94 | 1 | 24.01 | 0 | -1.30 | -1.30 |
| 2 | 3 | 12.54 | 1 | 63.94 | 1 | 24.01 | 0 | -0.88 | -0.88 |
| 2 | 4 | 18.28 | 1 | 63.94 | 1 | 24.01 | 0 | -1.40 | -1.40 |
| 3 | 1 | 0.00 | 1 | 62.81 | 0 | 19.43 | 1 | -0.67 | -0.67 |
| 3 | 2 | 3.03 | 1 | 62.81 | 0 | 19.43 | 1 | -0.66 | -0.66 |
| 3 | 3 | 9.34 | 1 | 62.81 | 0 | 19.43 | 1 | -1.14 | -1.14 |
| 3 | 4 | 17.51 | 1 | 62.81 | 0 | 19.43 | 1 | -2.22 | -2.22 |
| 4 | 1 | 0.00 | 0 | 65.04 | 1 | 25.08 | 0 | 0.44 | 0.44 |
| 4 | 2 | 3.13 | 0 | 65.04 | 1 | 25.08 | 0 | 0.78 | 0.78 |
| 4 | 3 | 11.49 | 0 | 65.04 | 1 | 25.08 | 0 | 1.29 | 1.29 |
| 4 | 4 | 17.14 | 0 | 65.04 | 1 | 25.08 | 0 | 1.83 | 1.83 |
| 5 | 1 | 0.00 | 0 | 66.80 | 0 | 17.69 | 1 | -0.92 | -0.92 |
| 5 | 2 | 2.98 | 0 | 66.80 | 0 | 17.69 | 1 | -0.33 | -0.33 |
| 5 | 3 | 10.17 | 0 | 66.80 | 0 | 17.69 | 1 | -1.65 | -1.65 |
| 5 | 4 | 18.41 | 0 | 66.80 | 0 | 17.69 | 1 |  | -2.70 |
| 6 | 1 | 0.00 | 1 | 69.08 | 1 | 23.53 | 0 | -1.75 | -1.75 |
| 6 | 2 | 3.38 | 1 | 69.08 | 1 | 23.53 | 0 | -1.99 | -1.99 |
| 6 | 3 | 9.98 | 1 | 69.08 | 1 | 23.53 | 0 | -0.94 | -0.94 |
| 6 | 4 | 17.22 | 1 | 69.08 | 1 | 23.53 | 0 | -0.62 | -0.62 |
| 7 | 1 | 0.00 | 1 | 71.24 | 0 | 7.99 | 1 | -1.11 | -1.11 |
| 7 | 2 | 2.68 | 1 | 71.24 | 0 | 7.99 | 1 | -0.55 | -0.55 |
| 7 | 3 | 13.72 | 1 | 71.24 | 0 | 7.99 | 1 |  | -1.24 |
| 7 | 4 | 17.60 | 1 | 71.24 | 0 | 7.99 | 1 |  | -2.63 |

**Appendix 3: SPM fitting algorithms and pseudo code**

A technical difficulty in using joint models can be getting them to actually run and produce results (known as “obtaining convergence”). Higher order models like SPMs require “iterative fitting” algorithms that start with initial parameter estimates and then iteratively improve them until a stopping criterion is reached (convergence). The biggest tip we can give is that the better the initial estimates are when starting the estimation procedure, the higher the chance that the fitting algorithms will converge and produce realistic estimates for the SPM. Here we provide pseudo-code of a stepwise approach that generally works in finding decent initial parameter estimates for SPM fitting. Depending on the size and structure of your data and the statistical package you use, fewer (or more) substeps may be necessary. Recall the basic form of the SPM:

Basic Shared Parameter Model:

1. *Longitudinal (LDA) submodel*:

Outcome over time = predictors + *latent characteristics*

Shared Information

1. *Censoring (EVENT) submodel*:

log(Event hazard) = baseline hazard + predictors

+ loading factors*(*latent characteristics)*

General SPM Pseudo-Code:

- Step 0: Conduct any **data** **curation** needed for the specific statistical package being used
- Step 1: Obtain **initial** **LDA** **estimates**: Fit the LDA mixed model **separately** and obtain initial estimates of:
  - LDA submodel regression parameters (β),
  - variance parameters (τ,σ), and
  - latent characteristics for each subject/id (empirical Bayes estimates: b*_0i_* , b*_1i_*, etc.)
- Step 2: Obtain **Initial** **EVENT** **estimates**: Fit the Weibull survival model **separately**, using the empirical Bayes estimates from Step 1 as additional predictors, and obtain initial estimates of:
  - EVENT submodel regression parameters (α),
  - Baseline hazard shape parameters (λ­_0_(t)), and
  - loading factors from latent characteristics (ρ)
- Step 3: **Improve Initial estimates**: Fit the **joint** **SPM**, initializing the parameter estimates from Steps 1 and 2 above, **but still using the calculated empirical Bayes estimates** from Step 1 as predictors in the EVENT submodel, to obtain better initial estimates of all sub-model parameters.
- Step 4: Obtain **final SPM estimates:** Fit the **joint** **SPM**, initializing all parameter estimates from Step 3. In this final step, do not include the calculated empirical Bayes estimates from Step 1 as predictors, but instead treat them as full latent constructs.

Each individual statistical package may require additional sub-steps within each of these general steps.
