# Supplementary material for: Reflection on modern methods: shared-parameter models for longitudinal studies with missing data
Source: Int J Epidemiol. 2021 Jun 11;50(4):1384–93. doi: 10.1093/ije/dyab086 (PMC8407871; doi:10.1093/ije/dyab086)

**Appendix 4: Stata Code for SPM**

Below we provide code for performing Joint / Shared Parameter Models (SPM) with Stata. We primarily used the **gsem** command. This package also allows for several other parameterizations for the joint model specification. It is also extendable to multiple longitudinal outcomes and multiple event outcomes and allows both Model-Based and Robust Standard Errors.

Pseudo-Code used for Stata:

**Step 0: Data curation**

**Step 1: Initial (separate) LDA estimates:**

1.1) Run gsem, get initial parameters (β, τ, σ, b*_0i_* , b*_1i_*,)

**Step 2: Initial (separate) EVENT estimates:**

2.1) Run gsem, get initial parameters (α, λ­_0_(t), ρ­_0_ , ρ­_1_) using (b*_0i_* , b*_1i_*) from step 1.1 as predictors

**Step 3: Improve Initial estimates:**

3.1) Run gsem, update initial parameters (β, τ, σ, α, λ­_0_(t), ρ­_0_ , ρ­_1_) using (b*_0i_* , b*_1i_*) from step 1.1 as predictors in the event submodel

3.2) IMPORTANT: rename the vector of parameter estimates to match what gsem will look for to initialize the final step

**Step 4:** **final SPM estimates:**

3.1) Run gsem to get final joint/SPM parameter estimates (β, τ, σ, α, λ­_0_(t), ρ­_0_ , ρ­_1_) initializing all parameter estimates from Step 3.2. In this final step, do not include the calculated empirical Bayes estimates (b*_0i_* , b*_1i_*) from Step 1.1 as predictors in the event submodel, but instead treat them as full latent constructs.

Stata code used for the analyses:

**************************************************************************

***** **Step 0: Data curation**

**************************************************************************

** get data;

import delimited "simdata.csv", clear

gen time = years/20 // scale time to 20 year effect

global adj male age0 // macro variable with adjustment vars for all models

**************************************************************************

***** **Step 1: Initial (separate) LDA estimates**

**************************************************************************

gsem (globz <- c.brainloss##c.time $adj U0[id]@1 c.time#U1[id]@1)

matrix lda = e(b) // store LDA beta & var estimates

capture drop _b0i _b1i

predict _b0i, latent(U0[id]) // EB random intercept ests

predict _b1i, latent(U1[id]) // EB random slope ests

**************************************************************************

***** **Step 2: Initial (separate) Event estimates**

**************************************************************************

gsem (demyears <- brainloss $adj _b0i _b1i, family(weibull, failure(dementia)))

matrix event = e(b) // store Event lambda, alpha & rho estimates

**************************************************************************

***** **Step 3: Improve initial estimates**

**************************************************************************

** Run submodels together, but use EB estimated latent effects in EVENT submodel

matrix init0 = lda, event // stack initial ests

gsem ///

(globz <- c.brainloss##c.time $adj U0[id]@1 c.time#U1[id]@1) ///

(demyears <- brainloss $adj _b0i _b1i, family(weibull, failure(dementia))) ///

, from(init0, skip)

matrix init1 = e(b) // store estimates

** IMPORTANT: names in the initial ests vector must match what gsem is looking for

global oldnames : colfullnames init1

global newnames = regexr("$oldnames","demyears:_b0i","demyears:U0[id]")

global newnames = regexr("$newnames","demyears:_b1i","demyears:U1[id]")

matrix colnames init1 = $newnames

**************************************************************************

***** **Step 4: Final SPM estimates**

**************************************************************************

** 4.1 Model Based SEs

gsem ///

(globz <- c.brainloss##c.time $adj U0[id]@1 c.time#U1[id]@1) ///

(demyears <- brainloss $adj U0[id]@r0 U1[id]@r1, fam(weib, fail(dementia))) ///

, from(init1, skip)

matrix spm_modelse = e(b) // store estimates

** 4.1 Huber-White semi-Robust Based SEs

gsem ///

(globz <- c.brainloss##c.time $adj U0[id]@1 c.time#U1[id]@1) ///

(demyears <- brainloss $adj U0[id]@r0 U1[id]@r1, fam(weib, fail(dementia))) ///

, from(spm_modelse, skip) vce(robust)


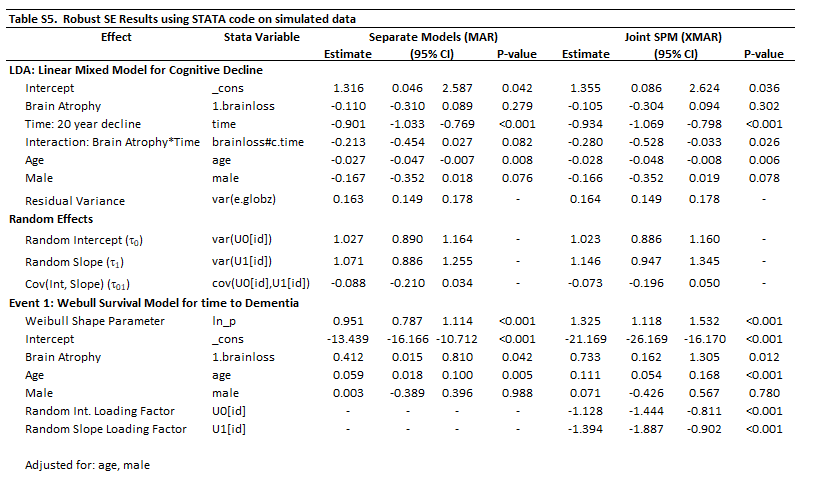

Supplement: dyab086_Supplementary_Data [file dyab086_supplementary_data.zip › ije-2020-03-0395-File007.docx]
