# Supplementary material for: Reflection on modern methods: shared-parameter models for longitudinal studies with missing data
Source: Int J Epidemiol. 2021 Jun 11;50(4):1384–93. doi: 10.1093/ije/dyab086 (PMC8407871; doi:10.1093/ije/dyab086)

**Appendix 5 - SAS Code for SPM**

Below we provide code for performing Joint / Shared Parameter Models (SPM) with SAS. We primarily used the **nlmixed** procedure. This procedure also allows for several other parameterizations for joint model specification. It is also extendable to multiple longitudinal outcomes and multiple event outcomes and allows both Model-Based and Robust Standard Errors.

Pseudo-Code used for SAS:

**Step 0: Data curation**

**Step 1: Initial (separate) LDA estimates:**

1.1) Run proc mixed, get initial parameters (β, τ, σ, b*_0i_* , b*_1i_*,)

**Step 2: Initial (separate) EVENT estimates:**

2.1) Run proc lifereg, get initial parameters (α, λ­_0_(t), ρ­_0_ , ρ­_1_) using (b*_0i_* , b*_1i_*) from step 1.1 as predictors

2.2) Reparametrize estimates to be on same scale as the event model specification used in proc nlmixed

**Step 3: Improve Initial estimates:**

3.1) Run proc nlmixed, update initial parameters (β, τ, σ, α, λ­_0_(t), ρ­_0_ , ρ­_1_) initializing parameter estimates from 1.1 and 2.2 above. Do not include the calculated (b*_0i_* , b*_1i_*) from Step 1.1 as predictors in the event submodel, but instead treat them as full latent constructs.

**Step 4:** **final SPM estimates:**

3.1) Run proc nlmixed one more time to get final joint/SPM parameter estimates (β, τ, σ, α, λ­_0_(t), ρ­_0_ , ρ­_1_) initializing all parameter estimates from Step 3.1. (This second run of proc nlmixed can help to ensure stable estimates, see: “Precision of Solution” notes in the “Computational Problems” section of the SAS proc nlmixed documentation)

SAS code used for the analyses:

**************************************************************************

***** **Step 0: Data curation**

**************************************************************************;

***** get data;

proc import datafile='1-data\simdata.csv' out=WORK._dat dbms=CSV; run;

proc sort data=_dat; by id visit; run;

data _dat; set _dat(where=(not missing(globz))); by id;

_first= first.id; *for non time-varying survival models, just use first obs;

time= years/20; *scale time var to 20 years;

brainloss_time = brainloss*time; *must create interactions by hand for nlmixed;

run;

**************************************************************************

***** **Step 1: Initial (separate) LDA estimates**

**************************************************************************;

proc mixed data=_dat noclprint method=ml cl covtest empirical ;

class id ;

model globz = brainloss time brainloss_time age0 male / s cl;

random intercept time / s subject=id type=un;

ods output solutionF=modelbased;

ods output CovParms=covp;

ods output SolutionR=sr(keep= effect id estimate); *save predicted random effect;

run;

***** format predicted random effects for merging;

proc transpose data=sr out=re prefix=estimate; by id; id effect; var estimate; run;

***** merge predicted random effect to data for proc lifereg;

data _dati; *call new data with predicted RE as _dati;

merge _dat Re(drop= _name_ rename=(estimateIntercept=u0i estimatetime=u1i));

by id; run;

**************************************************************************

***** **Step 2: Initial (separate) Event estimates**

**************************************************************************;

***** run Weibull survival model;

proc lifereg data=_dati(where=(_first=1));

model demyears*dementia(0)= brainloss age0 male u0i u1i / dist=weibull;

ods output ParameterEstimates= event_ests;

run;

***** reparameterize estimates (“-est/scale”) to be on same scale as proc nlmixed;

%let scale = 0.3126; *Note: 0.3126 is the scale from proc lifereg;

data event_ests; set event_ests(drop =DF);

if _N_ <7 then Estimate = -estimate/&scale;

run;

**************************************************************************

***** **Step 3: Improve initial estimates**

**************************************************************************;

proc nlmixed data=_dat empirical gconv=0;

*lda and event model initializing from step 1.1 and 2.2;

parms

b0=1.3162 b1=-0.1103 b2=-0.9009 b3=-0.2134 b4=-0.02718 b5=-0.1673

tau11=1.0273 tau21=-0.08817 tau22=1.0706 sigma=0.4042

a0=-18.0332 a1=0.5600 a2=0.09530 a3=0.05985 scale1=0.3126

r10=-0.9467 r11=-0.9212;

*LDA;

eta0 = b0 + b1*brainloss + b2*time + b3*brainloss_time + b4*age0 + b5*male ;

eta0i = eta0 + u0i + time*u1i; *LDA submodel linear predictor with RE;

resid = (globz-eta0i); *residual term;

s2 = sigma**2; *variance;

*LDA submodel likelihood specification;

if (abs(resid) > 1.3E100) or (s2 < 1e-12) then do; loglikeLDA = -1e20; end;

else do;loglikeLDA = -0.5*(1.837876 + resid**2 / s2 + log(s2)); end;

*EVENT;

if (_first) then do;

eta1 = a0 + a1*brainloss + a2*age0 + a3*male ;

eta1i = eta1 + r10*u0i + r11*u1i ; *Event submodel linear predictor with RE;

gam1 = 1/scale1; *Weibull survival scale parameter;

alph1 = exp(eta1i); *Weibull survival shape parameter;

*Event submodel likelihood specification;

loglikeEvent = (dementia=1)*(-alph1*(demyears**gam1) + log(gam1) + eta1i +

(gam1-1)*log(demyears)) + (dementia=0)*(-alph1*(demyears**gam1));

end;

else do; loglikeEvent=0; end;

*Joint SPM likelihood specification;

model _first ~ general(loglikeLDA + loglikeEvent);

random u0i u1i ~ normal([0, 0],[tau11,tau21,tau22]) subject=id; *RE distb;

ods output ParameterEstimates=init1; run;

**************************************************************************

***** **Step 4: Final SPM estimates**

**************************************************************************;

proc nlmixed data=_dat empirical gconv=0;

parms / data = init1; *parameter estimates initializing from step 3 ;

*LDA;

eta0 = b0 + b1*brainloss + b2*time + b3*brainloss_time + b4*age0 + b5*male ;

eta0i = eta0 + u0i + time*u1i; *LDA submodel linear predictor with RE;

resid = (globz-eta0i); *residual term;

s2 = sigma**2; *variance;

*LDA submodel likelihood specification;

if (abs(resid) > 1.3E100) or (s2 < 1e-12) then do; loglikeLDA = -1e20; end;

else do;loglikeLDA = -0.5*(1.837876 + resid**2 / s2 + log(s2)); end;

*EVENT;

if (_first) then do;

eta1 = a0 + a1*brainloss + a2*age0 + a3*male ;

eta1i = eta1 + r10*u0i + r11*u1i ; *Event submodel linear predictor with RE;

gam1 = 1/scale1; *Weibull survival scale parameter;

alph1 = exp(eta1i); *Weibull survival shape parameter;

*Event submodel likelihood specification;

loglikeEvent = (dementia=1)*(-alph1*(demyears**gam1) + log(gam1) + eta1i +

(gam1-1)*log(demyears)) + (dementia=0)*(-alph1*(demyears**gam1));

end;

else do;loglikeEvent=0; end;

*Joint SPM likelihood specification;

model _first ~ general(loglikeLDA + loglikeEvent);

random u0i u1i ~ normal([0, 0],[tau11,tau21,tau22]) subject=id; *RE distb;

ods output ParameterEstimates=spm; run;


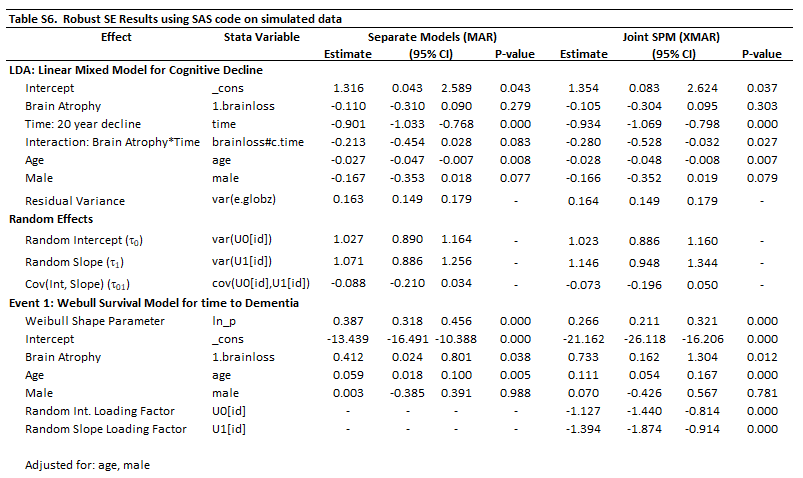

Supplement: dyab086_Supplementary_Data [file dyab086_supplementary_data.zip › ije-2020-03-0395-File008.docx]
