# Supplementary material for: Reflection on modern methods: shared-parameter models for longitudinal studies with missing data
Source: Int J Epidemiol. 2021 Jun 11;50(4):1384–93. doi: 10.1093/ije/dyab086 (PMC8407871; doi:10.1093/ije/dyab086)

**Appendix 6: R Code for SPM**

Below we provide code for performing Joint / Shared Parameter Models (SPM) with R. We used the package **JMBayes**. This procedure also allows for several other parameterizations for joint model specification. It also has additional alternative structures to connect the longitudinal model to event model, including: the expected value of the longitudinal measurement; the slope of the longitudinal measurement; interactions between covariates and the slope of the longitudinal measurement; and others. It is extendable to multiple longitudinal outcomes but currently only a single event outcome is available. We are unaware of an R package that accommodates SPMs with Robust standard errors; results for model-based SEs are shown below. Cox proportional hazards model using smoothers to approximate the baseline hazard was used for the event model as Weibull model is not available with the joint model implementation in JMBayes. The results using Weibull and Cox proportional hazards model are similar for the simulation data where the model assumptions are not violated.

Pseudo-Code used for R:

**Step 0: Data curation**

**Step 1: Initial (separate) LDA estimates:**

1.1) Run lme, get initial parameters (β, τ, σ, b*_0i_* , b*_1i_*,)

**Step 2: Initial (separate) EVENT estimates:**

2.1) Run coxph, get initial parameters (α, λ­_0_(t))

**Step 3:** **final SPM estimates:**

3.1) Run jointModelBayes to get final joint/SPM parameter estimates (β, τ, σ, α, λ­_0_(t), ρ­_0_ , ρ­_1_) initializing parameter estimates from Step 1.1 and 2.1.

R code used for the analyses:

#########################################################################

###### **Step 0: Data curation**

#########################################################################

##### load required packages

library(JMbayes)

library(tidyverse)

##### load the data set

Y <- read_csv("simdata.csv")

D <- Y %>%

mutate(

brainloss = recode_factor(brainloss, `0` = "No", `1` = "Yes"),

dementia = recode_factor(dementia, `0` = "NoDementia", `1` = "Dementia"),

male = recode_factor(male, `0` = "Female", `1` = "Male"),

time = years / 20,

id = as.factor(id) ) %>%

filter(!is.na(globz))

#########################################################################

##### **Step 1: Initial(separate) LDA estimates**

#########################################################################

lmeFit <- lme(globz ~ brainloss * time + age0 + male ,

random = ~ time | id,

data = D,

na.action = na.exclude)

summary(lmeFit)

#########################################################################

##### **Step 2: Initial(separate) EVENT estimates**

#########################################################################

##### use first observation to run survival model

Dsurv <- D %>% group_by(id) %>% filter(row_number() == 1) %>% ungroup()

##### cox proportional hazards survival model

wfit<- coxph(Surv(demyears,as.numeric(dementia))~brainloss+age0+male,

data = Dsurv,x=TRUE,na.action=na.exclude)

summary(wfit)

#########################################################################

##### **Step 3: Final SPM estimates**

#########################################################################

wfitJOINTBayes <- jointModelBayes(lmeFit, wfit,timeVar = "time", param = "shared-RE")

summary(wfitJOINTBayes)


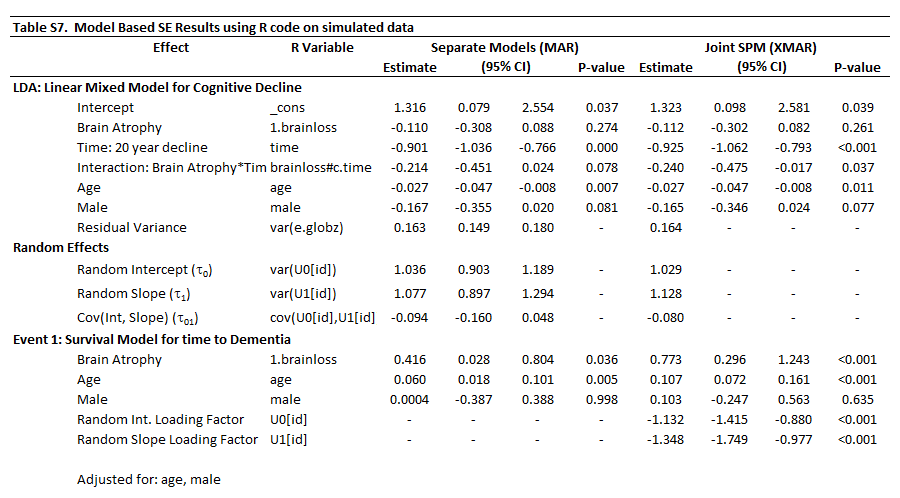

Supplement: dyab086_Supplementary_Data [file dyab086_supplementary_data.zip › ije-2020-03-0395-File009.docx]
