# Supplementary material for: Reflection on modern methods: shared-parameter models for longitudinal studies with missing data
Source: Int J Epidemiol. 2021 Jun 11;50(4):1384–93. doi: 10.1093/ije/dyab086 (PMC8407871; doi:10.1093/ije/dyab086)

**Appendix 7: SPM with Competing Risks of Dementia and Death (crSPM)**


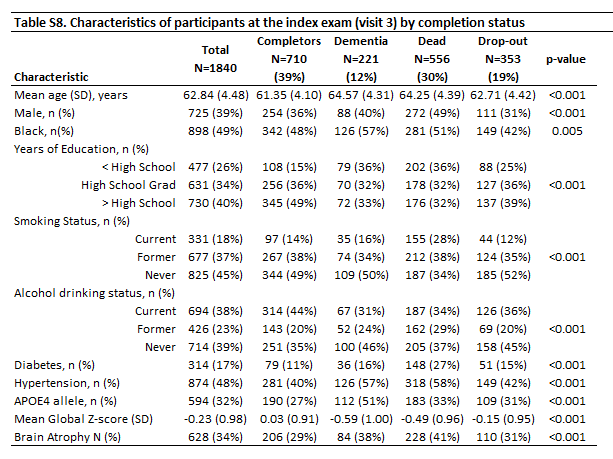


**crSPM (XMAR) model specification:**

Expanding the notation in Appendix 1, during follow-up, measurements on each participant may be lost due to Dementia, Death or simple dropout (right censored). Let *T_i_* = (*T_i_*, *K_i_*) be the times to Dementia, Death or censoring on subject *i*, with *K_i_* taking values {0=censoring, 1=dementia, 2=death}, and *T_ki_* = (*T_i_*, *K_i_*=k) indicating the censoring time is due to the *k*th reason. Throughout, right censoring is assumed independent of dementia and death and longitudinal outcomes are assumed independent of the censoring events after conditioning on the shared random effects. A joint, competing risks SPM (crSPM), extends the SPM by specifying:

Longitudinal GLMM submodel

1. GlobalZ*_ij_*|**b***_i_* = Xβ*_ij_* + b*_0i_* + b*_1i_*⋅(yrs) + e*_ij_*
2. b*_oi_* ~ N(0, τ*_0_*^2^); b*_1i_* ~ N(0, τ*_1_*^2^); Cov(b*_oi_*,b*_1i_*) = τ*_01_*
3. e*_ij_*|**b***_i_* ~ N(0, σ^2^)

Dementia Event Submodel:

1. log{H(T_1_*_i_*|**b***_i_*)} = log{H_0_(T_1_*_i_*)} + α_11_(atrophy) + **α_1_**⋅(**adj**) + ρ_10_b_0i_ + ρ_11_b_1i_
2. T_1_*_i_*|**b***_i_* ~ Weibull

Death Event Submodel:

1. log{H(T_2_*_i_*|**b***_i_*)} = log{H_0_(T_2_*_i_*)} + α_21_(atrophy) + **α_2_**⋅(**adj**) + ρ_20_b_0i_ + ρ_21_b_1i_
2. T_2_*_i_*|**b***_i_* ~ Weibull

Where atrophy is indicator for brain atrophy and **α_1_**⋅(**adj**) & **α_2_**⋅(**adj**) represent vectors of additional regression parameters for each event submodel and the design matrix corresponding to the adjustment covariates specified above. Robust standard errors were employed.


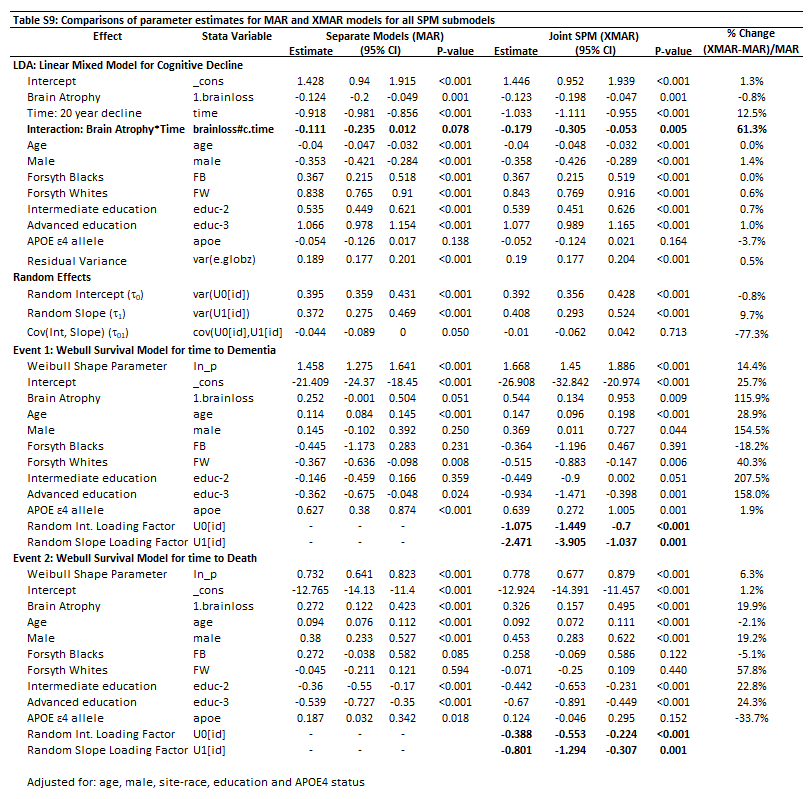

Supplement: dyab086_Supplementary_Data [file dyab086_supplementary_data.zip › ije-2020-03-0395-File010.docx]
